# Supplementary material for: Effects of Royal Jelly on Gut Dysbiosis and NAFLD in db/db Mice
Source: Nutrients. 2023 May 31;15(11):2580. doi: 10.3390/nu15112580 (PMC10255852; doi:10.3390/nu15112580)
Supplement: Supplementary file 1 [file nutrients-15-02580-s001.zip › Supplementary Figure S1.pdf]

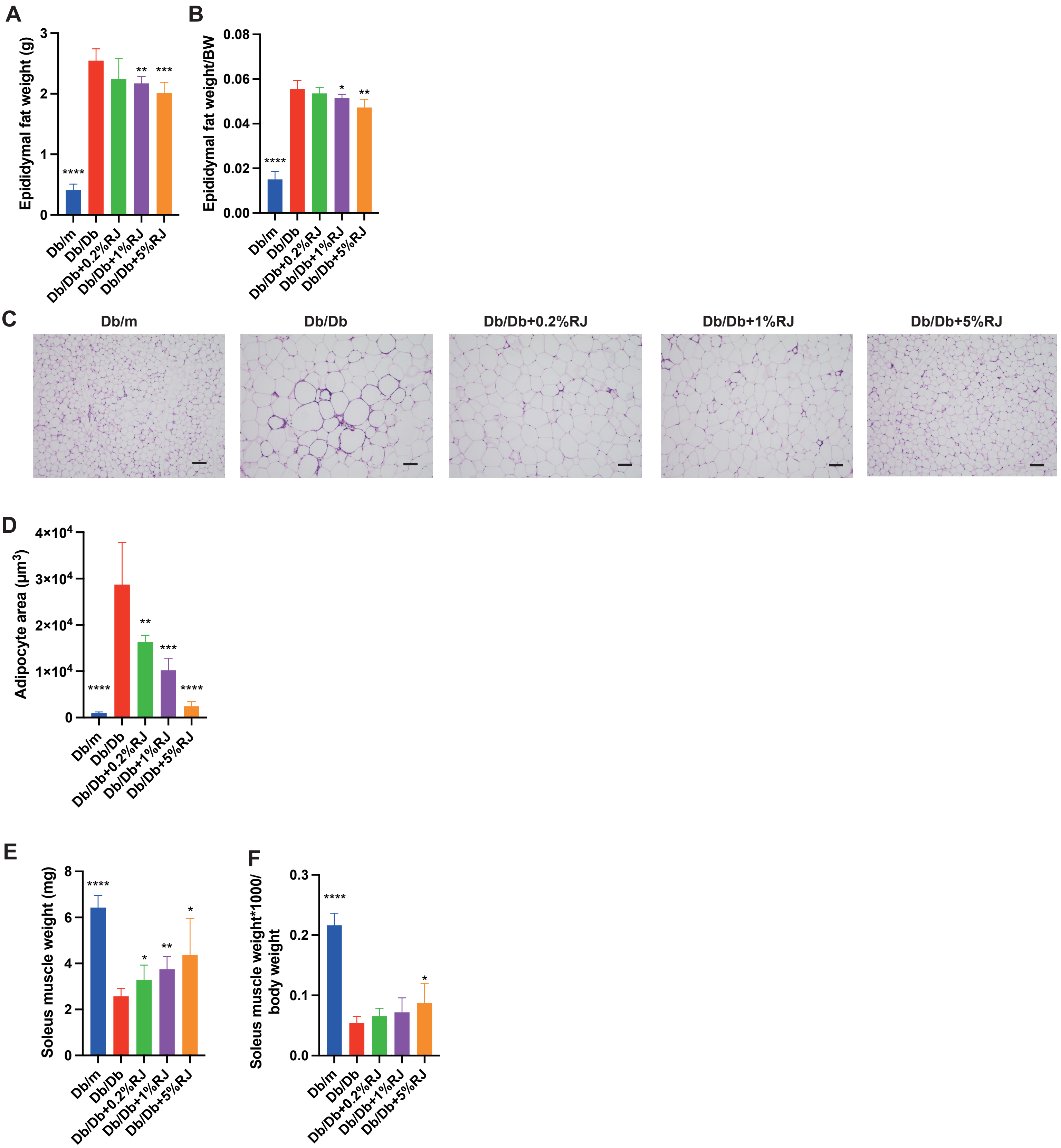

Supplementary Figure S1. db/db mice fed with royal jelly showed lower visceral fat accumulation and higher skeltal muscle mass

(A and B) Absolute and relative weights of epididymal fat (n = 6).

(C) Representative images of hematoxylin & eosin-stained epididymal fat section.

(D) Adipocyte area (n=6). (E and F) Absolute and relative solues muscle mass (n=6)

Data are represented as the mean ± SD values. Data were analyzed using 1-way ANOVA with Holm-Šídák’ s multiple-comparisons test. *p < 0.05, **p < 0.01, ***p < 0.001, ****p < 0.0001.
