# Supplementary material for: Effects of Royal Jelly on Gut Dysbiosis and NAFLD in db/db Mice
Source: Nutrients. 2023 May 31;15(11):2580. doi: 10.3390/nu15112580 (PMC10255852; doi:10.3390/nu15112580)
Supplement: Supplementary file 1 [file nutrients-15-02580-s001.zip › Supplementary Table S1.pdf]

Supplementary Table S1. The comparison among six groups.

	PA	PA+Decanoic acid	PA+Decenoic acid	PA+2-Decenodioc aicd	PA+Sebacic acid
Oil red O stains	†	†‡	†‡	†‡§	†‡‖
FASN	†	†‡	‡§	†‡§¶	†‡§¶‖
SCD1	†	†‡	†‡§	†‡¶	†‡§‖
COL1A1	†	†‡	†‡§	†‡¶	‡§‖

The analyses among six groups were performed by one-way ANOVA with Holm–Šídák's multiple-comparisons test: †, p<0.05 versus Ctrl; ‡, p<0.05 versus PA; §, p<0.05 versus PA+Decanoic acid; ¶, p<0.05 versus PA+Decenoic acid; ‖, p<0.05 versus PA+2-Decenodioc acid.
